# Cocoa Spread with Grape Seed Oil and Encapsulated Grape Seed Extract: Impact on Physical Properties, Sensory Characteristics and Polyphenol Content

**DOI:** 10.3390/foods11182730

**Published:** 2022-09-06

**Authors:** Ivana Lončarević, Jovana Petrović, Nemanja Teslić, Ivana Nikolić, Nikola Maravić, Biljana Pajin, Branimir Pavlić

**Affiliations:** 1 Faculty of Technology, University of Novi Sad, Bulevar cara Lazara 1, 21000 Novi Sad, Serbia; 2 Institute of Food Technology, University of Novi Sad, Bulevar cara Lazara 1, 21000 Novi Sad, Serbia

**Keywords:** cocoa spread, grape seed oil, grape seed extract, rheology, thermal properties, polyphenols, sensory characteristics

## Abstract

The aim of this study was to utilize grape pomace, as a polyphenol-rich by-product of wine production, in the manufacture of enriched cocoa spread. The formulation of the cocoa spread has been modified by substitution of refined sunflower oil with cold-pressed grape seed oil. The spread with grape seed oil (Cg) was further enriched with grape seed extract encapsulated on maltodextrins (E), where 10% and 15% of E was added to Cg obtaining the samples Cg10 and Cg15. The results showed an increase in volume-weighted mean in spread samples, from 19.17 μm in Cg to 19.71 μm in Cg10 and 21.04 μm in Cg15. Casson yield stress and Casson viscosity significantly (*p* ˂ 0.05) increased from 16.41 Pa and 1.58 Pa·s in Cg to 29.45 Pa and 5.70 Pa·s in Cg15 due to the reduction of the fat-phase content in enriched spreads. The addition of E had no significant effect on the melting temperature (T_peak_) of the enriched spreads, while increasing the amount of E significantly (*p* ˂ 0.05) increased their hardness. The incorporation of grape seed oil in the cocoa spread formulation contributed to an increase in total polyphenols and flavonoids. Moreover, the addition of 10% and 15% of E to Cg resulted in approximately 1.5× and 2× higher content of phenolic compounds in Cg10 and Cg15 compared to control spread with sunflower oil (Cs). Flavonoids increased from 0.43 mg CE/g in Cs to 0.74 mg CE/g in Cg 10 and 1.24 mg CE/g in Cg15. Encapsulates positively affected sensory characteristics of enriched spread samples by reducing their grape seed oil aroma and sweetness.

## 1. Introduction

Consumers are increasingly seeking food that will prevent chronic illness and optimize their health. Global market size of functional food products is estimated at USD 74 billion [1] and it is estimated to grow by approximately 10% per year [2]. The fact is that the food industry is producing large amounts of waste of which management and disposal is a serious environmental problem. Nowadays, a new process for the controlled disposal of waste is converting these materials into other bio-products. One of the main by-products generated by the wine industry is grape pomace, which is composed of grape seeds, skin and stem remains [3]. Over 0.2 kg of grape pomace is produced per 1 kg of grapes, which contains approximately 25% seeds. Bearing in mind that world grape production in 2020 was 78,034,332 t [4], more than 0.3 kg of solid side-stream waste is generated per kg of freshly mashed grape fruit [5]. Several studies have shown that grape seed (dry base) contains about 35% crude fiber, 29% extractable components, 7–20% oil, 11% protein, 3% minerals and 7% moisture [3].

Serbia has a moderate continental climate and favorable geological conditions suitable for grape growing [6]. During the 2020 vintage, 160,307 t of grapes were produced in Serbia [4]; thus, a considerable amount of grape waste is generated during the vinification process. Ultimately, given the significance of grape pomace, utilization of waste for producing oil as a source of functional compounds is becoming a promising field.

One way of utilizing grape waste is the production of grape seed oil [7]. The interest in grape seed oil as a functional food product has increased, especially because of its high levels of phenolic compounds (the main polyphenols being catechins, epicatechins, trans-resveratrol and procyanidin B1), vitamin E (ranging from 1 to 53 mg per 100 g of oil, and 148–358 α-tocopherol equivalents), unsaturated fatty acids (totaling 85–90%, of which 66.0–75.3% is linoleic acid) and phytosterols. On an industrial scale grape seed oil can be obtained using either an organic solvent or mechanical techniques. Cold-pressing is a method that involves no heat or chemical treatment and hence may retain more health-beneficial components [8]. However, supercritical fluid extraction could be used as an excellent green-and-clean method for recovery of grape seed oil and could provide significant improvement in terms of yield and product quality [9]. On the other hand, ultrasound-assisted extraction (UAE) with moderately polar solvents, such as aqueous ethanol, could be efficiently used for isolation of grape seed polyphenols. Numerous studies have confirmed the tremendous advantages of UAE over conventional solid–liquid extraction, providing the polyphenol-rich extracts with higher yield and reducing the solvent and time consumption [10,11].

While the oil and protein are found in grape seed endosperm, phenolic compounds and crude fiber are mainly present in the seed coat and can be also extracted. Extracted active compounds can be protected from environmental conditions (light, moisture, oxygen) by the encapsulation process [12]. This procedure provides a physical barrier between the active compounds, adverse environmental conditions and the food matrix. The economical and flexible process of microencapsulation involves spray drying which converts liquids into powders. This provides for easier handling, storage and transportation of encapsulates and facilitates its uniform mixing into food formulations [13].

Cocoa spread is a confectionery product that can be nutritionally enriched since it is based on powdered sugar, vegetable fat, cocoa powder, milk powder and other ingredients like hazelnut paste, emulsifier lecithin and aroma. Unlike chocolate, cocoa spread products do not contain cocoa butter, but rather contain cheaper vegetable fats and may also contain vegetable oil to improve its spreadability. The quality of cocoa spread is strongly influenced by the behavior and oxidative stability of its fat phase, which often amounts to over 30% of the final product. The fat phase greatly affects the production process of the spread product as well as its sensory acceptance, stability and price. Moreover, it is of great importance to also focus on its functional properties which largely depend on the type of fat used [14,15]. Aydemir [16] used refined super palm olein-fraction oil, anhydrous vegetable margarine, hazelnut oil, coconut oil, anhydrous milk fat, olive oil and sunflower seed oil in cocoa hazelnut cream production while Guzmán et al. [17] used sesame oil and sesame oil cake in the development of cocoa spread. Aydemir [18] investigated the effects of different fat/oil and chestnut contents on the quality of chestnut cream. Aydemir et al. [19] used glucose syrup to substitute 2.5%, 5%, 10% and 20% (*w*/*w*) of sugar in cocoa hazelnut cream. Moreover, Bascuas et al. [20] used hydrocolloid-based oleogels in chocolate spreads. 

In some available literature, the authors utilized grape waste in the production of ice cream and chocolate spread. Akca and Akpinar [21] improved the functional properties of probiotic ice cream with the addition of grape seed pulp powder and grape seed oil. In another study, Acan et al. [22] used dried grape pomace in the chocolate spread instead of various amounts of powdered sugar and milk. The investigation of Sagdic et al. [23] involved the effect of grape seed extract on the sensorial and antioxidant properties of probiotic ice cream.

However, no scientific literature sources have so far published any results that involve testing the quality of cocoa-based confectionery products formulated with grape seed oil and encapsulated grape pomace. Thus, the aim of this study was to utilize the grape waste in cocoa spread production by investigating the effects of grape seed oil and encapsulated grape pomace on the rheological properties, melting behavior, textural characteristics, sensory properties and polyphenol content of cocoa spread.

## 2. Materials and Methods

### 2.1. Materials

The materials used in this work included powdered sugar, cocoa powder, milk powder (all purchased from confectionery factory “Jaffa”, Crvenka, Serbia), refined sunflower oil and palm oil (both purchased from oil factory “Dijamant”, Zrenjanih, Srbija), cold-pressed grape seed oil and red grape seeds (“Kovačević Winery D.O.O.”, Irig, Serbia) and lecithin (produced in oil factory “Sojaprotein”, Bečej, Srbija). The fatty-acid profile of grape seed oil consisted of palmitic (7.20–7.93 g/100 g), palmitoleic (0.12–0.17 g/100 g), oleic (13.39–18.47 g/100 g), linoleic (68.61–74.15 g/100 g), γ-linolenic (0–0.25 g/100 g), α-linolenic (0.39–0.66 g/100 g) and heneicosanoic (0.19–0.34 g/100 g) acids [9].

### 2.2. Plan of Experiments

In the first stage of the experiment the control sample of the cocoa spread was created with the addition of refined sunflower oil (sample Cs) while in the second stage the sunflower oil was replaced with cold-pressed grape seed oil to create cocoa spread with grape seed oil (sample Cg).

The composition of the cocoa spread includes: powdered sugar 50%, palm fat 24%, refined sunflower oil or cold-pressed grape seed oil 6%, cocoa powder 8%, milk powder 11.5% and lecithin 0.5%.

Cocoa spread was produced in a laboratory ball mil (“Masino Produkt”, Crvenka, Serbia) with a capacity of 5 kg. The temperature in the ball mill was 40 °C, with a speed of 50 rpm. After 90 min of milling, the spread was dosed into sterile plastic cups.

In the third stage of the experiment, cocoa spread mass with grape seed oil was substituted with the addition of 10% (sample Cg10) and 15% (sample Cg15) of encapsulated grape seed extract (E) obtained from the cake remaining after oil extraction. After the cocoa spread with grape seed oil was produced in the laboratory ball mill under the same conditions, the spread mass was dosed in a plastic bucket; the mass of the spread was then measured. The specific mass of E was added and the spread mass was poured into a laboratory homogenizer with a capacity of 5 kg (“Masino Produkt”, Crvenka, Serbia). The spread was homogenized with encapsulates for 30 min at 40 °C and dosed into sterile plastic cups.

### 2.3. Preparation of Grape Seed Encapsulate

Grape seeds were milled in a domestic blender (Bosch, MMB21P0R/01, Germany) and mean particle size of the sample was determined by sieving through the vibro-sieve set (CISA Cedaceria Industrial, Spain). The mean particle size for the red and white grape seeds was 0.578 mm. Supercritical fluid extraction (SFE) was utilized in order to remove oil from the sample according to the previously optimized procedure [9]. Defatted grape seed samples were used as the raw material for the recovery of polyphenols and production of powder extract, i.e., grape seed encapsulate.

Ultrasound-assisted extraction (UAE) with previously optimized conditions was used for production of liquid extract. UAE was conducted in a bath sonicator (EUP540A, Euinstruments, France) at a frequency of 40 KHz, temperature of 56 °C, ethanol concentration of 53% and extraction time of 29 min [24]. Extraction of 30 g of a raw material with 300 mL of extraction solvent (1:10, *w*/*v*) in 500 mL glass flasks was done in order to prepare a sufficient volume of liquid extract for the spray drying (4 L).

Liquid extracts obtained by UAE were dried using a spray-drying process in the pilot Anhydro spray-dryer plant (APV Anhydro AS, Denmark) according to the procedure previously described by Lim et al. [25]. The feed was preheated to 50 °C on a heating plate with magnetic stirrer. Processing temperatures for spray drying were 150 °C for the inlet temperature and 70–75 °C for the outlet temperature. Liquid feed was transferred to the drying chamber using a laboratory peristaltic pump (FH100 Series, Thermo Scientific, Waltham, MA, USA) with 1.36 L/h flow rate. Maltodextrin was added as carrier agent with a 50:50 (*m*/*m*) ratio to grape seed solid concentration to the liquid extract.

### 2.4. Methods

#### 2.4.1. Particle Size Distribution of Encapsulated Grape Seed Extract and Cocoa Spread Samples

The particle size distribution of the encapsulates and cocoa spread samples was determined by the Mastersizer 2000 laser diffraction particle size analyzer (Malvern Instruments, Malvern, UK). The encapsulates were dispersed in air using a Scirocco dispersion unit, while spreads were dispersed in sunflower oil using the Hydro 2000 μP dispersion unit. The dispersed samples were added to the sunflower oil at ambient temperature until an adequate obscuration was obtained (10–20%). The results were quantified as a volume-based particle size distribution by means of the Mastersizer 2000 software (version number 5.60, Malvern, UK).

#### 2.4.2. Rheological Properties of Cocoa Spread Samples

The rheological properties of the samples were determined by a rotational rheometer, the Rheo Stress 600 (Haake, Karlsruhe, Germany), at a temperature of 40 ± 1 °C [26]. The flow curves were determined by applying the method of the hysteresis loop using a concentric cylinder system (sensor Z20 DIN). The shear rate was first increased from 0 s^−1^ to 60 s^−1^ over a period of 180 s, then kept constant for 60 s at the max. speed of 60 s^−1^ before being reduced from 60 s^−1^ to 0 s^−1^ within 180 s.

#### 2.4.3. Thermal Properties of Cocoa Spreads

The melting properties of cocoa spread samples were measured using a Differential Scanning Calorimeter DSC Q100 (TA Instruments). Approximately 5 mg of samples were weighed into aluminum pans. The hermetically sealed pans were then heated from 10 to 50 °C (5 °C/min) in the DSC using an empty aluminum pan as reference. The onset temperature (T_onset_), peak maximum (T_peak_), conclusion temperature (T_end_) and enthalpy of melting (H_melt_) were automatically calculated after integrating the melting peaks using data analysis software (TA Instruments, New Castle, DE, USA) [27].

The cocoa spread samples were heated from 10 °C to 50 °C with a heating rate of 5 °C per minute using a Differential Scanning Calorimeter DSC 910, Thermal Analyzer 990 and Dynamic Mechanical Analyzer (TA Instruments, New Castle, DE, USA). 5 mg of the spread sample was measured into aluminum pans and the pierced covers were sealed in place. An empty, hermetically sealed aluminum pan was used as a reference.

The melting properties of the spread samples were defined using DSC parameters: onset temperature (T_onset_), peak temperature (T_peak_) and conclusion temperature (T_end_). T_onset_ is the temperature at which a specific crystal form starts to melt, T_peak_ is the temperature at which melting rate is the greatest and T_end_ is the temperature at which melting ends [28].

#### 2.4.4. Textural Properties of Cocoa Spreads

Textural characteristics of cocoa spread samples were analyzed using a Texture Analyzer TA.XT Plus (Stable Micro System, Godalming, UK). The maximum force (kg) and work of shear (kg·s) were measured during the analysis by penetration at an ambient temperature of 21 ± 1 °C according to the Chocolate Spread–SPRD2_SR_PRJ method. Each sample was placed in the cone sample holder and excess sample was scraped off with a knife. Then, the filled cone sample holder was placed in the base holder and the 45º cone probe with a diameter of 38 mm penetrated the samples at 3 mm/s. The obtained maximum force indicates the hardness of the cocoa spread, and the size below the surface of the force-dependence curve from the time represents the work of shear in the applied force.

The maximum force (kg) and work of shear (kg·s) were measured during the analysis. The maximum force is defined as the hardness at the specific penetration depth, while the size below the surface of the obtained force-dependence curve (F) from the time (t) represents the work of shear in the applied force.

#### 2.4.5. Sensory Analyses

The sensory panel was comprised of 9 assessors (5 women and 4 men), all food technologists. They were selected according to the guidelines of the ISO 8586:2012 standards [29]. Ethical review and approval were waived for this study since the participation was voluntary. All data were anonymous. Informed consent was obtained from all subjects involved in the study. The attribute list was obtained through discussion with the panel leader. Five cocoa spread samples representing a wide range of sensory characteristics were presented to the assessors. Through a consensus process they agreed on the most adequate attributes to fully describe the dynamics of the sensory characteristics of the samples. Next, sample evaluation cards with the panelist’s list of definitions of attributes and their lowest and highest intensity were prepared. 

The final list of attributes included 10 attributes covering appearance, aroma, flavor and texture to be used in the examination. The panelists were asked to rate the intensity, using a 7-point scale, of each attribute [30]: color uniformity (1: non-uniform to 7: uniform); glow (1: matte to 7: oil migration); hardness, i.e., the ease with which samples could be spread on a biscuit (1: extremely soft to 7: extremely hard); graininess, i.e., the proportion of small solids between teeth during chewing (1: smooth to 7: grainy); melting, i.e., the time it takes for spread to transform into a liquid in the mouth (1: quick to 7: slow); cocoa flavor, or characteristic taste related to the presence of cocoa (1: poorly expressed to 7: very expressed); grape seed oil flavor (1: non-existent to 7: very expressed); sweetness, or characteristic taste related to the presence of sucrose (1: poorly expressed to 7: very expressed); and grape seed oil taste (1: non-existent to 7: very expressed). 

The tests were conducted in the sensory laboratory at the Faculty of Technology Novi Sad, in individual cabinets illuminated with white light, designed in accordance with ISO 8589 [31]. The cocoa spread samples were served at room temperature (21 °C) on three-digit-numbered plastic cups. Mineral water at room temperature and diced peeled apple were served between sample servings. The average point number was calculated for each sample.

#### 2.4.6. Polyphenol Content and Antioxidant Activity in Cocoa Spread Samples

Prior to spectrophotometric analysis, cocoa spread samples were defatted according to a modified method published by Belščak et al. [32]. First, 8 g of spread was mixed with 20 mL of hexane in an Erlenmeyer flask and placed in an orbital shaker for 10 min (150 rpm). The sample was then centrifuged at 3000 rpm for 10 min. Supernatant was separated from sediment. The sample-defatting step was repeated 3 times. Once obtained, the defatted solid part was air-dried for 24 h. Phenols were extracted from the defatted sample with 10 mL of 70% methanol using an ultrasound bath (EUP540A, Euinstruments, France) for 30 min at room temperature. The sample was then centrifuged at 3000 rpm for 10 min and the supernatant was utilized for further analysis. The methanolic extracts obtained were tested in terms of total phenolic content (TP), total flavonoid content (TF), ABTS^+^ and DPPH radical-scavenging activity and ferric reducing antioxidant power according to methods published by Pavlić et al. [10]. All spectrophotometric analyses were performed in duplicate.

#### 2.4.7. Statistical Analysis

All experiments were performed in triplicate except for the sensory analysis including 9 panelists and the spectrophotometric assays, which were performed for 6 repetitions. The obtained results were statistically tested using the ANOVA method and the means were compared by one-factor analysis at variance with subsequent comparisons by Duncan’s test at a significance level at 0.05 using Statistica 13.3 software (TIBCO Software Inc., Paolo Alto, CA, USA, 2016).

## 3. Results and Discussion

### 3.1. The Impact of Grape Seed Oil and Encapsulated Grape Seed Extract on Particle Size Distribution and Rheological Properties of Cocoa Spreads

Since cocoa spread presents a complex multiphase system of different solid particles (sugar, cocoa powder, milk powder, etc.) dispersed in a continuous fat phase, its particle size distribution, viscosity and consistency largely depend on the milling process during production as well as on the type of fat used [21]. Consumer acceptance strongly depends primarily on appearance and taste, but also very much on mouth feel, which itself mainly depends on the particle size and the viscosity of the molten chocolate or cocoa spread mass [33].

**Figure 1:**
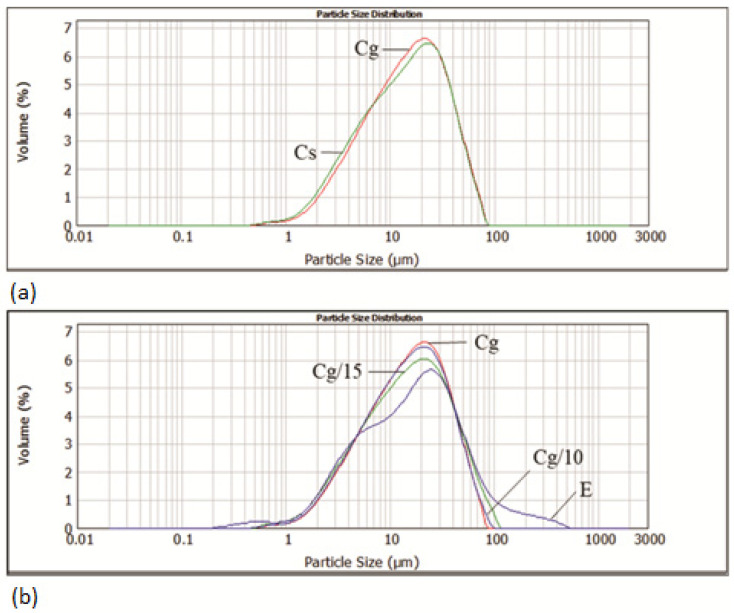
Particle size distribution of: (**a**) cocoa spreads with sunflower (Cs) and grape seed (Cg) oil; (**b**) encapsulate (E), Cg and enriched spreads Cg10 and Cg15.

Figure 1 shows the impact of grape seed oil and E on the particle size distribution of cocoa spread samples.

Replacing sunflower oil with grape seed oil leads to a barely noticeable difference in the particle size distribution curve. Encapsulated bioactive components (E) also have a very desirable particle size distribution as an additive in the production of cocoa spread products, but also contain particles in the range of 100–500 µm which leads to an increase in the volume mean diameter D [4,3] of samples Cg10 and Cg15 (Table 1).

Cocoa spread with grape seed oil has significantly (*p* ˂ 0.05) higher values for d (0.1) and d (0.5) compared to cocoa spread with sunflower oil, while their values for d (0.9) are not significantly different. However, the volume mean diameter D [4,3] of Cg is significantly (*p* ˂ 0.05) higher (19.60 µm) compared to Cs (19.17 µm). Addition of 10 and 15% of E to spread with grape seed oil lowers the values of d (0.1) and d (0.5) in Cg10 and Cg15 and at the same time significantly (*p* ˂ 0.05) increases the values of d (0.9) in accordance with the added amount. An increase in the value of this parameter affects the increase in D [4,3] in samples Cg10 (19.71 µm) and significantly (*p* ˂ 0.05) in Cg15 (21.04 µm). Despite small differences in D [4,3], all samples have mean diameters in the desired interval (15–30 μm) that provides the appropriate rheological and sensory characteristics of chocolate and cocoa-related products [33].

**Figure 2:**
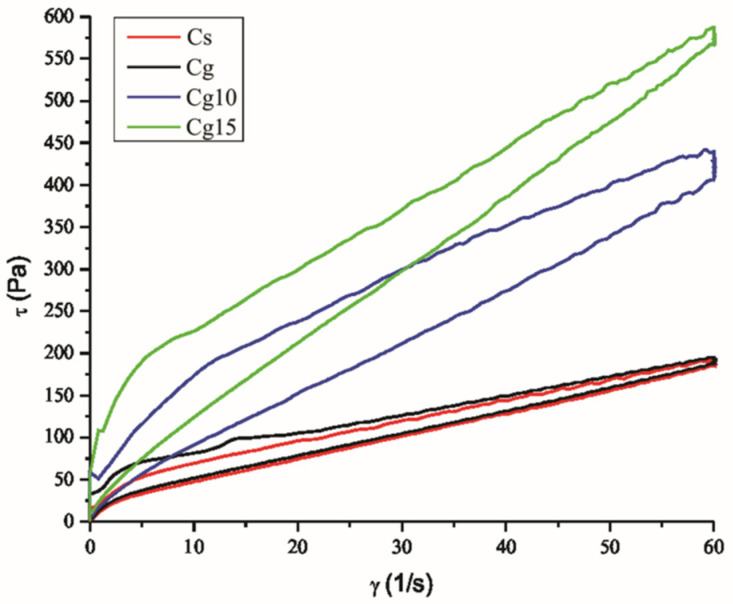
Flow curves of cocoa spreads with sunflower oil (Cs) and grape seed oil (Cg) and enriched samples with 10% (Cg10) and 15% (Cg15) of encapsulated grape seed extract.

Cocoa spread, like chocolate liqueur, exhibits thixotropic properties characterized by yield stress and plastic flow [34]. Figure 2 shows the influence of grape seed oil and E on the flow properties of cocoa spread samples at production temperature (40 °C).

The cocoa spread samples exhibit a thixotropic type of flow. It can be noticed from Figure 2 that the substitution of sunflower with grape seed oil has an impact on the flow of cocoa spread mass at lower shear rates (shown by the ascending curve) while the viscosity is very similar at higher shear rates and when reducing shear rate from 60 1/s to 0 1/s. Also, it is very noticeable that the addition of encapsulated grape seed bioactive components to cocoa spread with grape seed oil significantly increases the viscosity in accordance with the added amount. This is also indicated by the rheological parameters shown in Table 2. The flow curves of the cocoa spreads were fitted using the Casson model to get the following parameters: Cason yield stress (Pa) and Casson viscosity (Pa·s).

Although the substitution of sunflower with grape seed oil in the cocoa spread formulation significantly (*p* ˂ 0.05) increases the value of Casson yield stress, it doesn’t have the significant impact on Casson viscosity. The higher yield stress of Cg and the difficult flow at lower shear rates also influences the significantly (*p* ˂ 0.05) higher value of the thixotropic curve area of this sample compared to the control Cs. The addition of 10 and 15% of E to cocoa spread with grape seed oil significantly (*p* ˂ 0.05) increases the values of Casson yield stress, Casson viscosity and thixotropic curve area in accordance with the added amount of E. The fact is that the fat phase coats the solid particles in molten cocoa spread and reduces resistance to flow; thus, reducing fat amount through the addition of E causes a higher incidence of particle interaction that makes initial flow more difficult and increases the viscosity of enriched spread samples in accordance with the added amount.

### 3.2. The Impact of Grape Seed Oil and Encapsulated Grape Seed Extract on Thermal and Textural Properties of Cocoa Spread

Fasina et al. [35] investigated melting characteristics of different vegetable oils. The results showed that grape seed oil has lower values of all DSC parameters compared to sunflower oil. However, the substitution of sunflower oil with grape seed oil in the cocoa spread formulation doesn’t have significant influence on T_onset_ and T_peak_ values, while it significantly (*p* ˂ 0.05) decreases the value of T_end_ in Cg compared to Cs (Table 3). The addition of 10% and 15% of E to Cg doesn’t significantly affect the values of T_onset_ and T_peak_, while 15% of E significantly (*p* ˂ 0.05) increases the value of T_end_.

The replacement of sunflower oil with grape seed oil also doesn’t have a significant effect on hardness and work of shearing. However, the decrease in the amount of the fat phase in enriched cocoa spreads caused by the addition of 10 and 15% of E to Cg leads to a significant increase (*p* ˂ 0.05) in hardness and work of shear values in Cg10 and Cg15 compared to the control sample Cg, whereas the values of textural parameters of Cg15 are significantly (*p* ˂ 0.05) higher than values of Cg10.

### 3.3. The Impact of Grape Seed Oil and Encapsulated Grape Seed Extract on Sensory Characteristics of Cocoa Spread

The values of color uniformity, glow, hardness, graininess, cocoa flavor and sweetness do not differ significantly between Cs and Cg (Table 4).

However, Cg has significantly (*p* < 0.05) highest mean values for grape seed oil flavor and grape seed oil taste compared to all examined samples. Sagdic et al. [23] concluded that the addition of grape seed oil to probiotic ice cream increased the functional properties of enriched ice cream without causing a negative sensory effect. This was not the case with the addition of grape seed oil to cocoa spread, where the panelists concluded that the flavor and taste have been made slightly worse compared to the control sample containing sunflower oil. However, the addition of both amounts of E to Cs reduces the expression of grape seed oil flavor and grape seed oil taste, even significantly (*p* < 0.05) between Cg 10 and Cg15. On the other hand, both amounts of E significantly negatively affect glow, hardness and graininess in Cs10 and Cs15, with significant difference (*p* < 0.05) between their values. Furthermore, only 15% of E causes significant (*p* < 0.05) reduction of melting and lowers cocoa flavor in Cs15 compared to Cs and Cs10.

### 3.4. The Impact of Grape Seed Oil and Encapsulated Grape Seed Extract on Polyphenol Content of Cocoa Spread

**Figure 3:**
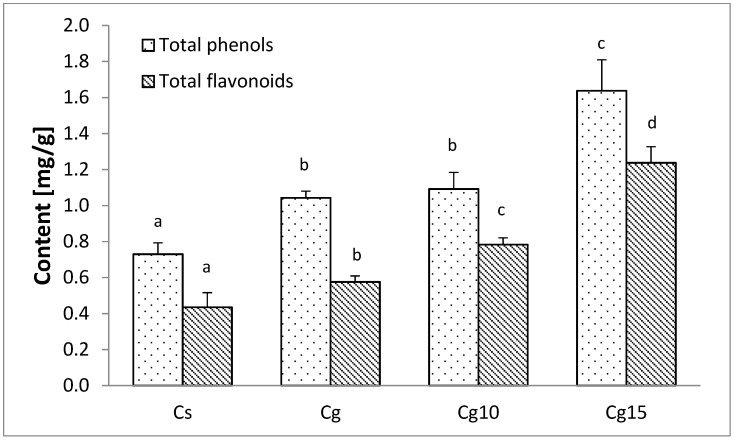
Total phenol and total flavonoid content of cocoa spreads with sunflower oil (Cs) and grape seed oil (Cg) and enriched samples with 10% (Cg10) and 15% (Cg15) of encapsulated grape pomace. Values represent the mean value of 6 measurements. Values followed by the same letter within the same column are not significantly different (*p* > 0.05) according to Duncan’s test.

Waste fractions of food processing are promising sources of polyphenols (i.e., red grape pomace), which are in high demand because of their bioactivities [36]. Red grape pomace consists mainly of grape seeds and skin. This winemaking by-product contains a wide range of phenolic compounds such as phenolic acids, flavonols, flavanols, anthocyanidins, stilbenes, tannins and their derivatives [37,38]. Thus, adding encapsulated red grape pomace would increase polyphenolic content of the cocoa spread (Figure 3).

The addition of encapsulated grape seed extract in amounts of 10 and 15% results in ~1.5× and ~2× higher content of phenolic compounds in Cg10 and Cg15 sample when compared to the control sample (Cs). Encapsulates significantly enhance total flavonoid content of Cg10 (0.738 mg CE/g; ~2× fold) and Cg15 (1.238 mg CE/g; ~3× fold) to an even greater extend when compared to control cocoa spread (0.435 mg CE/g). This suggests that, indeed, grape seed extract encapsulates are carriers of valuable bioactive compounds in Cg10 and Cg15 samples. Thus, consumption of polyphenol- and particularly flavonoid-enriched spreads could partly satisfy the required daily intake of polyphenols. According to certain research flavonoid intake of 500 mg/day or higher could be associated with lower risk of cardiovascular events and/or mortality [39]. Thus, polyphenol-enhanced diets seems to be linked with health benefits in humans and could be used as a valid tool for the prevention of various chronic diseases [39]. Addition of plant encapsulates or powders in confectionary products is not an entirely novel approach. As it was reported in a recent study, blackberry juice encapsulates were used to enrich white chocolate with polyphenols [40]. Results indicated that chocolate with added blackberry juice encapsulates (80 g/kg) had significantly higher polyphenolic content (145.86 g/100 g) compared to control chocolate (40.75 g/100 g), while pleasant fruity flavor and reduced sweetness improved the sensory attributes of chocolate with encapsulates. Furthermore, usage of grape seed powder extract resulted in a higher polyphenol bioavailability when grape-powder-enriched white chocolate (in saliva = 31.8 mg/kg; in gastric juice = 130.9 mg/kg; in intestinal juice = 154.2 mg/kg) was in vitro digested compared to control (in saliva = 0.38 mg/kg; in gastric juice = 195.1 mg/kg; in intestinal juice = 154.2 mg/kg) [41]. However, to the best of our knowledge, usage of winemaking by-products as polyphenol carriers in cocoa spread has not been studied before.

### 3.5. The Impact of Grape Seed Oil and Encapsulated Grape Seed Extract on Antioxidant Activity of Cocoa Spread

**Figure 4:**
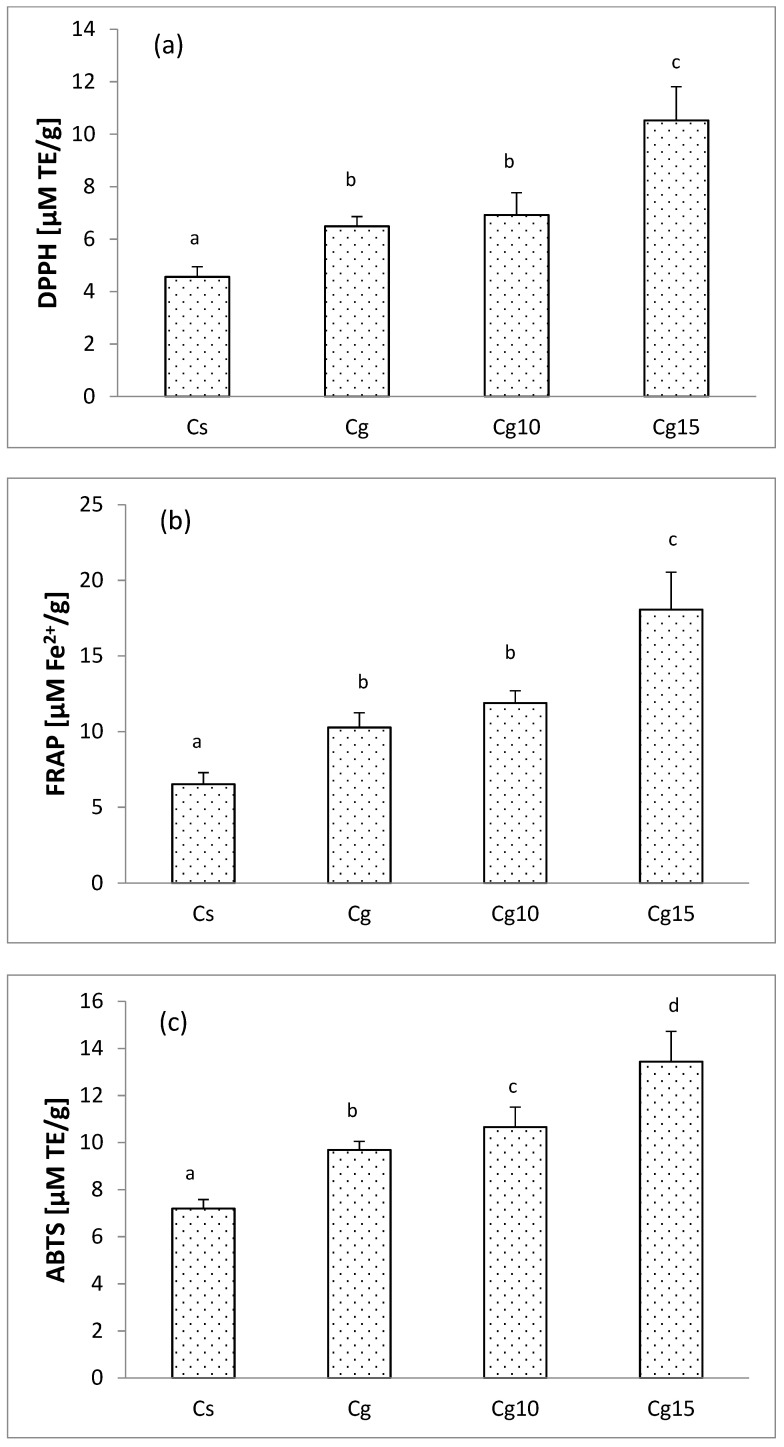
Antioxidant activity determined with (**a**) DPPH assay (**b**) ABTS assay and (**c**) FRAP assay in cocoa spreads with sunflower oil (Cs) and grape seed oil (Cg) and enriched samples with 10% (Cg10) and 15% (Cg15) of encapsulated grape seed extract. Values represent the mean value of 6 measurements. Values followed by the same letter within the same column are not significantly different (*p* > 0.05) according to Duncan’s test.

Since encapsulated grape seed extract is a carrier of phenolic compounds which are well-known antioxidant agents, it is rather expected that antioxidant activity of Cg10 and Cg15 would be significantly higher compared to control (Cs) (Figure 4). Such an observation was concluded for all in vitro antioxidant assays performed.

Substitution of refined sunflower oil with grape seed oil also increases antioxidant power in cocoa spread. It could be speculated that the higher quantity of polyphenols and/or other antioxidants naturally present in cold-pressed oils [42] was retained in the defatted residue of Cg compared to Cs. Regardless, products rich in fatty content such as cocoa spread are prone to lipid oxidation. This process is undesirable in confectionary products since it negatively alters the organoleptic properties via the appearance of rancid flavor. One viable strategy to prevent lipid oxidation and to extend the shelf-life of cocoa spreads is addition of polyphenol-rich plant extracts. Indeed, addition of up to 3% of raspberry and blueberry extract significantly increased antioxidant potential and lipid oxidative stability of chocolate [43] supporting viability of such strategy.

## 4. Conclusions

From a sensory point of view, the replacement of refined sunflower oil with cold-pressed grape seed oil in the formulation of cocoa spread has no significant effect on the color and glow of the spread, nor on the texture and melting properties, but, at the same time, contributes to a very pronounced grape seed oil aroma which is not common in this type of confectionery product. However, the addition of encapsulated grape seed extract has a positive effect on the flavor and taste of the enriched spread samples by reducing the grape seed oil aroma and sweetness. Grape seed oil also increases the total polyphenols and flavonoids of the cocoa spread, as the addition of 10% and 15% of encapsulated grape seed extract to cocoa spread with grape seed oil further increases total polyphenols (1.5-fold in Cg10 and 2-fold in Cg15 compared to the control spread with sunflower oil) and total flavonoids. Future research will aim to increase the share of grape seed oil in the cocoa spread formulation that includes encapsulated seed extract and grape seed oil in order to increase the fat-phase content in the enriched cocoa spread samples, thereby reducing their viscosity and hardness.

## Figures and Tables

**Table 1:** Particle size parameters of Cs, Cg, E and enriched cocoa spread samples Cg10 and Cg15.

Sample	Particle Size Parameters (µm)			
	d (0.1)	d (0.5)	d (0.9)	D [4,3]
Cs	3.56 ± 0.06 ^b^	14.85 ± 0.12 ^a^	41.44 ± 0.36 ^a^	19.17 ± 0.10 ^a^
Cg	3.96 ± 0.09 ^c^	15.32 ± 0.04 ^c^	41.48 ± 0.32 ^a^	19.60 ± 0.11 ^b^
E	3.30 ± 0.03 ^a^	17.06 ± 0.13 ^e^	62.65 ± 0.44 ^d^	30.67 ± 0.12 ^d^
Cg10	3.85 ± 0.04 ^c^	15.09 ± 0.15 ^b^	42.40 ± 0.28 ^b^	19.71 ± 0.14 ^b^
Cg15	3.55 ± 0.07 ^b^	15.24 ± 0.04 ^b,c^	46.94 ± 0.16 ^c^	21.04 ± 0.06 ^c^

Values represent the mean value of 3 measurements. Values followed by the same letter within the same column are not significantly different (*p* > 0.05) according to Duncan’s test.

**Table 2:** Rheological parameters of cocoa spread samples.

Sample	Casson Yield Stress (Pa)	Casson Viscosity (Pa·s)	Thixotropic Curve Area (Pa/s)
Cs	9.90 ± 0.25 ^a^	1.86 ± 0.18 ^a^	1127 ± 19.85 ^a^
Cg	16.41 ± 0.43 ^b^	1.58 ± 0.13 ^a^	1482 ± 23.11 ^b^
Cg10	23.90 ± 0.65 ^c^	4.11 ± 0.33 ^b^	5352 ± 77.65 ^c^
Cg15	29.45 ± 0.82 ^d^	5.70 ± 0.31 ^c^	6085 ± 86.52 ^d^

Values represent the mean value of 3 measurements. Values followed by the same letter within the same column are not significantly different (*p* > 0.05) according to Duncan’s test.

**Table 3:** Thermal and textural parameters of cocoa spread samples.

Sample	Thermal Properties			Textural Parameters	
	T_onset_ (°C)	T_peak_ (°C)	T_end_ (°C)	Hardness (kg)	Work of Shear (kg·s)
Cs	31.62 ± 0.32 ^a^	35.15 ± 0.21 ^a,b^	42.82 ± 0.29 ^b^	3.88 ± 0.18 ^a^	3.02 ± 0.22 ^a^
Cg	32.06 ± 0.17 ^b^	35.48 ± 0.38 ^b,c^	42.19 ± 0.21 ^a^	3.71 ± 0.24 ^a^	2.99 ± 0.18 ^a^
Cg10	32.32 ± 0.25 ^b^	35.72 ± 0.21 ^c^	42.47 ± 0.29 ^a,b^	8.81 ± 0.35 ^b^	7.58 ± 0.31 ^b^
Cg15	32.47 ± 0.36 ^b^	35.41 ± 0.21 ^b,c^	42.83 ± 0.28 ^b^	20.31 ± 0.71 ^c^	20.39 ± 0.64 ^c^

Values represent the mean value of 3 measurements. Values followed by the same letter within the same column (within the values for model system or white chocolate) are not significantly different (*p* > 0.05) according to Duncan’s test.

**Table 4:** Sensory characteristics of chocolate samples.

Sensory Parameter	Sample			
	Cs	Cg	Cg10	Cg15
Color uniformity	6.72 ± 0.44 ^c^	6.28 ± 0.26 ^b,c^	6.17 ± 0.35 ^a,b^	5.78 ± 0.44 ^a^
Glow	3.94 ± 0.30 ^c^	3.80 ± 0.25 ^c^	2.85 ± 0.24 ^b^	1.89 ± 0.22 ^a^
Hardness	2.18 ± 0.21 ^a^	2.30 ± 0.15 ^a^	4.08 ± 0.22 ^b^	6.32 ± 0.35 ^c^
Graininess	1.61 ± 0.22 ^a^	1.78 ± 0.26 ^a^	2.39 ± 0.42 ^b^	4.33 ± 0.25 ^c^
Melting	1.72 ± 0.26 ^a^	2.39 ± 1.20 ^a,b^	2.83 ± 0.25 ^b,c^	4.55 ± 0.30 ^d^
Cocoa flavor	5.22 ± 0.36 ^c,d^	5.11 ± 0.22 ^b,c^	4.77 ± 0.26 ^b^	3.59 ± 0.49 ^a^
Grape seed oil flavor	1.00 ± 0.00 ^a^	6.02 ± 0.15 ^d^	5.50 ± 0.22 ^c^	4.84 ± 0.24 ^b^
Sweetness	6.04 ± 0.25 ^c^	5.80 ± 0.40 ^c^	4.76 ± 0.25 ^b^	4.10 ± 0.22 ^a^
Grape seed oil taste	1.00 ± 0.00 ^a^	6.32 ± 0.30 ^d^	5.25 ± 0.26 ^c^	4.22 ± 0.21 ^b^

Values followed by the same letter within the same row are not significantly different (*p* > 0.05) according to Duncan’s test.

## Data Availability

Data are contained within the article.

## References

[1] Illanes A. (2016). Lactose: Production and upgrading. Lactose-Derived Prebiotics: A Process Perspective.

[2] Kähkönen M.P., Hopia A.I., Vuorela H.J., Rauha J.P., Pihlaja K., Kujala T.S., Heinonen M. (1999). Antioxidant activity of plant extracts containing phenolic compounds. J. Agric. Food Chem..

[3] Yang C., Shang K., Lin C., Wang C., Shi X., Wang H., Li H. (2021). Processing technologies, phytochemical constituents, and biological activities of grape seed oil (GSO): A review. Trends Food Sci. Technol..

[4] FAOSTAT. http://www.fao.org/faostat/en/#data.

[5] Duba K.S., Fiori L. (2015). Supercritical CO_2_ extraction of grape seed oil: Effect of process parameters on the extraction kinetics. J. Supercrit. Fluids.

[6] Ruml M., Vuković A., Vujadinović M., Djurdjević V., Ranković-Vasić Z., Atanacković Z., Sivčev B., Marković N., Matijašević S., Petrović N. (2012). On the use of regional climate models: Implications of climate change for viticulture in Serbia. Agric. For. Meteorol..

[7] Dabetic N.M., Todorovic V.M., Djuricic I.D., Antic Stankovic J.A., Basic Z.N., Vujovic D.S., Sobajic S.S. (2020). Grape Seed Oil Characterization: A Novel Approach for Oil Quality Assessment. Eur. J. Lipid Sci. Technol..

[8] Garavaglia J., Markoski M.M., Oliveira A., Marcadenti A. (2016). Grape Seed Oil Compounds: Biological and Chemical Actions for Health. Nutr. Metab. Insights.

[9] Dimić I., Teslić N., Putnik P., Kovačević D.B., Zeković Z., Šojić B., Mrkonjić Ž., Čolović D., Montesano D., Pavlić B. (2020). Innovative and conventional valorizations of grape seeds from winery by-products as sustainable source of lipophilic antioxidants. Antioxidants.

[10] Pavlić B., Teslić N., Vidaković A., Vidović S., Velićanski A., Versari A., Radosavljević R., Zeković Z. (2017). Sage processing from by-product to high quality powder: I. Bioactive potential. Ind. Crops Prod..

[11] Mrkonjić Ž., Rakić D., Olgun E.O., Canli O., Kaplan M., Teslić N., Zeković Z., Pavlić B. (2021). Optimization of antioxidants recovery from wild thyme (*Thymus serpyllum* L.) by ultrasound-assisted extraction: Multi-response approach. J. Appl. Res. Med. Aromat. Plants.

[12] Fang Z., Bhandari B. (2010). Encapsulation of polyphenols—A review. Trends Food Sci. Technol..

[13] Böger B.R., Georgetti S.R., Kurozawa L.E. (2018). Microencapsulation of grape seed oil by spray drying. Food Sci. Technol..

[14] Lončarević I., Pajin B., Petrović J., Zarić D., Sakač M., Torbica A., Lloyd D.M., Omorjan R. (2016). The impact of sunflower and rapeseed lecithin on the rheological properties of spreadable cocoa cream. J. Food Eng..

[15] Lončarević I., Pajin B., Sakač M., Zarić D., Rakin M., Petrović J., Torbica A. (2016). Influence of Rapeseed and Sesame Oil on Crystallization and Rheological Properties of Cocoa Cream Fat Phase and Quality of Final Product. J. Texture Stud..

[16] Aydemir O. (2019). Utilization of different oils and fats in cocoa hazelnut cream production. J. Food Process. Preserv..

[17] Guzmán R.E., Gómez J.D., Chocrón S. (2020). Potential use of Sesame (*Sesamum indicum* L.) oil and sesame oil cake in the development of spreadable cocoa cream. Am. J. Food Sci. Nutr..

[18] Aydemir O., Atalar İ. (2019). Functionality of chestnut and fat/oil contents in cocoa chestnut cream production—A new product development. J. Food Process Eng..

[19] Aydemir O., Beşir A., Aden H.M. (2021). Textural and rheological characteristics of cocoa hazelnut cream partially substituted with glucose syrup. Eur. Food Sci. Eng..

[20] Bascuas S., Espert M., Llorca E., Quiles A., Salvador A., Hernando I. (2021). Structural and sensory studies on chocolate spreads with hydrocolloid-based oleogels as a fat alternative. LWT.

[21] Akca S., Akpinar A. (2021). The Effects of Grape, pomegranate, Sesame Seed Powder and Their Oils on Probiotic Ice Cream: Total phenolic contents, antioxidant activity and probiotic viability. Food Biosci..

[22] Acan B.G., Kilicli M., Bursa K., Toker O.S., Palabiyik I., Gulcu M., Yaman M., Gunes R., Konar N. (2021). Effect of grape pomace usage in chocolate spread formulation on textural, rheological and digestibility properties. LWT.

[23] Sagdic O., Ozturk I., Cankurt H., Tornuk F. (2012). Interaction Between Some Phenolic Compounds and Probiotic Bacterium in Functional Ice Cream Production. Food Bioprocess Technol..

[24] Ghafoor K., Choi Y.H., Jeon J.Y., Jo I.H. (2009). Optimization of ultrasound-assisted extraction of phenolic compounds, antioxidants, and anthocyanins from grape (Vitis vinifera) seeds. J. Agric. Food Chem..

[25] Lim K., Ma M., Dolan K.D. (2011). Effects of Spray Drying on Antioxidant Capacity and Anthocyanidin Content of Blueberry By-Products. J. Food Sci..

[26] IOCCC (2000). Viscosity of Cocoa and Chocolate Products. Anal. Method.

[27] Aidoo R.P., Afoakwa E.O., Dewettinck K. (2015). Rheological properties, melting behaviours and physical quality characteristics of sugar-free chocolates processed using inulin/polydextrose bulking mixtures sweetened with stevia and thaumatin extracts. LWT—Food Sci. Technol..

[28] Afoakwa E.O., Paterson A., Fowler M., Vieira J. (2008). Relationship between rheological, textural and melting properties of dark chocolate as influenced by particle size distribution and composition. Eur. Food Res. Technol..

[29] (2012). Sensory analysis—General Guidelines for the Selection, Training and Monitoring of Selected Assessors and Expert Sensory Assessors.

[30] (2002). Sensory Analysis—Methodology—Evaluation of Food Product by Methods of Using Scales.

[31] (2007). Sensory Analysis-General Guidance for the Design of Test Rooms.

[32] Belščak A., Komes D., Horžić D., Ganić K.K., Karlović D. (2009). Comparative study of commercially available cocoa products in terms of their bioactive composition. Food Res. Int..

[33] Bolenz S., Holm M., Langkrär C. (2014). Improving particle size distribution and flow properties of milk chocolate produced by ball mill and blending. Eur. Food Res. Technol..

[34] Pajin B., Dokić L., Zarić D., Šoronja-Simović D., Lončarević I., Nikolić I. (2013). Crystallization and rheological properties of soya milk chocolate produced in a ball mill. J. Food Eng..

[35] Fasina O.O., Craig-Schmidt M., Colley Z., Hallman H. (2008). Predicting melting characteristics of vegetable oils from fatty acid composition. LWT—Food Sci. Technol..

[36] Hellwig V., Gasser J. (2020). Polyphenols from waste streams of food industry: Valorisation of blanch water from marzipan production. Phytochem. Rev..

[37] Lingua M.S., Fabani M.P., Wunderlin D.A., Baroni M.V. (2016). In vivo antioxidant activity of grape, pomace and wine from three red varieties grown in Argentina: Its relationship to phenolic profile. J. Funct. Foods.

[38] Ricci A., Parpinello G.P., Palma A.S., Teslić N., Brilli C., Pizzi A., Versari A. (2017). Analytical profiling of food-grade extracts from grape (*Vitis vinifera* sp.) seeds and skins, green tea (*Camellia sinensis*) leaves and Limousin oak (*Quercus robur*) heartwood using MALDI-TOF-MS, ICP-MS and spectrophotometric methods. J. Food Compos. Anal..

[39] Del Bo C., Bernardi S., Marino M., Porrini M., Tucci M., Guglielmetti S., Cherubini A., Carrieri B., Kirkup B., Kroon P. (2019). Systematic Review on Polyphenol Intake and Health Outcomes: Is there Sufficient Evidence to Define a Health-Promoting Polyphenol-Rich Dietary Pattern?. Nutrients.

[40] Lončarević I., Pajin B., Fišteš A., Tumbas Šaponjac V., Petrović J., Jovanović P., Vulić J., Zarić D. (2018). Enrichment of white chocolate with blackberry juice encapsulate: Impact on physical properties, sensory characteristics and polyphenol content. LWT.

[41] Altınok E., Kurultay S., Konar N., Toker O.S., Kopuk B., Gunes R., Palabiyik I. (2022). Utilising grape juice processing by-products as bulking and colouring agent in white chocolate. Int. J. Food Sci. Technol..

[42] Bjelica M.S., Vujasinovi V., Rabrenovi B., Dimi S., Vujasinović V., Rabrenović B., Dimić S. (2019). Some Chemical Characteristics and Oxidative Stability of Cold Pressed Grape Seed Oils Obtained from Different Winery Waste. Eur. J. Lipid Sci. Technol..

[43] Kaur M., Kumar S., Bhat Z.F., Naqvi Z., Jayawardena R. (2022). The impact of raspberry and blueberry extract on the microbial and lipid oxidative stability of calcium and chicken protein fortified composite chocolate. J. Food Process. Preserv..

